# Melatonin rescues cell respiration impaired by hypoxia/reoxygenation in aortic endothelial cells and affects the mitochondrial bioenergetics targeting the F_1_F_O_-ATPase

**DOI:** 10.1016/j.redox.2025.103605

**Published:** 2025-03-20

**Authors:** Cristina Algieri, Chiara Bernardini, Antonia Cugliari, Silvia Granata, Fabiana Trombetti, Patrycja Anna Glogowski, Micaela Fabbri, Giampaolo Morciano, Gaia Pedriali, Paolo Pinton, Salvatore Nesci

**Affiliations:** a Department of Veterinary Medical Sciences, University of Bologna, Ozzano Emilia, 40064, Italy; b Health Sciences and Technologies-Interdepartmental Center for Industrial Research (CIRI-SDV), Alma Mater Studiorum—University of Bologna, 40126, Bologna, Italy; c IRCCS Neuromed, Pozzilli, 86077, Italy; d Translational Research Center, Maria Cecilia Hospital GVM Care & Research, Cotignola, 48033, Italy; e Department of Medical Sciences, Laboratory for Technologies of Advanced Therapies (LTTA), University of Ferrara, Ferrara, 44121, Italy; f Department of Biosciences, Biotechnology and Environment, University of Bari Aldo Moro, Bari, 70125, Italy

**Keywords:** F_1_F_O_-ATPase, H/R injury, ROS production, Melatonin, Mitochondrial dysfunction, Mitochondrial permeability transition pore

## Abstract

Melatonin is evaluated as a potential molecular therapy to counteract mitochondrial dysfunction caused by hypoxia/reoxygenation (H/R) in aortic endothelial cells (pAECs). The mitochondrial permeability transition pore (mPTP) opening undergoes a desensitizing action coupled with a reduction of superoxide anion production in mitochondria treated with melatonin. The effect on mPTP has been attributed to the direct interaction of melatonin with the hydrophilic F_1_ domain of Ca^2+^-activated F_1_F_O_-ATPase. Mutual exclusion analysis highlights an overlapping binding site between melatonin and the specific F_1_ inhibitor NBD-Cl. The results are corroborated by melatonin inhibition of ATPase activity of the purified F_1_ domain in the presence of Ca^2+^, but not in the presence of natural cofactor Mg^2+^. Moreover, the impairment of bioenergetics parameters in pAECs metabolism and the increase of oxidative stress arising by H/R injury have been rescued in cells protected by melatonin treatment.

## Highlights

• F_1_-ATPase is a mitochondrial molecular target of melatonin.
• Melatonin exerts a protective effect on mPTP opening and mitochondrial SOX production.
• Protective effect of melatonin on H/R injury-induced damage.

## Graphical abstract

**Figure:**
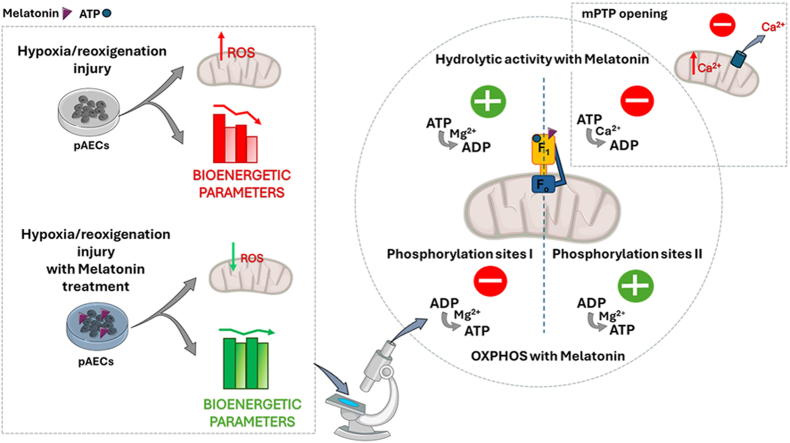

## 1 Introduction

Cardiovascular diseases (CVDs) are responsible for most deaths worldwide. Although the mortality rate is decreasing, the prevalence of CVDs is still too high [1]. The beneficial effects of melatonin in the treatment of various human diseases, including CVDs, have been widely studied. Melatonin is an indole hormone derived from serotonin via the tryptophan-serotonin biosynthetic pathway produced locally in various tissues but mainly by the pineal gland. Melatonin synthesis starts from tryptophan through the action of tryptophan hydroxylase, which is transformed into 5-hydroxytryptophan which, in turn, is converted into serotonin. The latter is acetylated by arylalkylamine *N*-acetyltransferase to *N*-acetylserotonin and converted into melatonin by acetylserotonin *O*-methyltransferase [2]. Pineal synthesis of melatonin is governed by the suprachiasmatic nucleus, which is synchronized to the light-dark cycle by the retinohypothalamic tract, favouring its synthesis at night in the dark. Melatonin has a sleep-promoting role, which is why melatonin concentrations increase when the light fades, peak during darkness, and decrease when exposed to light to promote wakefulness [3]. However, melatonin also promotes immune regulation and modulation of pituitary and adrenal hormones [4]. It exerts its direct functions via a receptor-dependent signalling pathway or an indirect function as a free radical scavenger. The chemical messenger of melatonin involves interaction with specific cellular receptors widely distributed in various organs such as the retina, brain, kidneys, gastrointestinal tract, skin, and the immune, endocrine, reproductive, and cardiovascular systems. The receptors are G-protein coupled and thus modulate the activities of adenylate cyclase, guanylate cyclase and phospholipase C. Consequently, calcium and potassium fluxes into the cell [5]. Some of the receptor-related effects of melatonin include modulation of the activity of enzymes involved in cellular protection against damage caused by reactive oxygen species (ROS) and reactive nitrogen species (RNS), although it also has a known inhibitory effect on nitric oxide synthase and lipoxygenase responsible for the synthesis of superoxide anion [6]. It is one of the most powerful natural antioxidants. Melatonin can directly chelate ROS and RNS, but might also mobilize the intracellular antioxidant enzyme system [7]. These actions protect many biological molecules from oxidative damage and suppress the development of serious degenerative disorders such as neuronal, cardiac and tumour diseases [8]. Reactive species contribute to the pathogenesis of cardiac ischemic reperfusion injury. However, melatonin, exerting its ROS scavenging function in the mitochondria where it is found in large quantities [9,10], has beneficial effects in ischemic heart disease by preventing myocardial reperfusion injury [3,[11], [12], [13], [14], [15]]. Its benefits are therefore associated with the reduction of oxidative stress, modulation of metabolic activity, regulation of cytokine production and prevention of cell apoptosis [16,17].

Mitochondria are essential organelles responsible for cellular energy supply via oxidative phosphorylation activity but are also the main site of ROS generation. Mitochondrial and cellular function impairment induced in the presence of ROS can be controlled with antioxidant compounds. Melatonin synthesis and metabolism can occur in mitochondria [18], which tend to contain higher levels of melatonin than other compartments, such as the cytosol. Melatonin metabolites have been detected in mitochondria and cytochrome *c* is thought to participate in this melatonin metabolism [19]. Therefore, it could be a potential mitochondrial protector considering its antioxidant properties [20]. Indeed, melatonin may improve mitochondrial function by stimulating respiratory chain activity, mainly complexes I, III and IV, and increases mitochondrial ATP production in both normal and pathological conditions [21]. Although melatonin's cardiovascular actions against ischemia/reperfusion (I/R) injury are evident [22,23], the mechanism is still unclear. In this study, we evaluate the protective effect of melatonin on mitochondrial dysfunction that sustains hypoxia/reoxygenation (H/R) injury, focusing on the altered cellular energy metabolism caused by impaired oxidative phosphorylation leading to ATP depletion, increased oxidative stress and induction of mitochondrial permeability transition pore (mPTP) opening [24].

Mitochondrial F_1_F_O_-ATPase is a bifunctional enzyme that supports cellular ATP synthesis/hydrolysis when activated by the natural cofactor Mg^2+^. However, the enzyme is considered the main component responsible for mPTP formation when associated with the Ca^2+^ cation [25,26]. The Ca^2+^-activated F_1_F_O_-ATPase and consequently mPTP opening is a phenomenon strongly related to acute I/R injury [27]. mPTP formation is triggered in conditions of oxidative stress, mitochondrial [Ca^2+^] overload and altered phosphate levels during ischemia [28].

The results provided may lead to improvements in the field of melatonin-mediated altered mitochondrial bioenergetics forming the basis for its future use in therapy in the field of cardiovascular diseases.

## 2 Materials and methods

### 2.1 Mitochondrial isolation

Immediately after slaughter, hearts from adult swine (*Sus scrofa domesticus*) were collected at a local abattoir and transported to the laboratory within 2 h in ice buckets at 0–4 °C. After the removal of fat and blood clots, approximately 30–40 g of heart tissue was rinsed in ice-cold washing Tris-HCl medium A (0.25 M sucrose, 10 mM Tris (hydroxymethyl)-aminomethane (Tris), pH 7.4). The tissue was chopped into fine pieces with scissors, gently dried on blotting paper, and weighed. The chopped tissue was homogenized in a medium B (0.25 M sucrose, 10 mM Tris (pH 7.4 with HCl), 1.0 mM EDTA (free acid), 0.5 mg/mL bovine serum albumin (BSA)), at a ratio of 10 mL medium B per 1 g of fresh tissue. After a gentle breakup by Ultraturrax T25, to obtain the mitochondrial fraction by stepwise centrifugation (Sorvall RC2-B, rotor SS34), the homogenate was centrifuged at 1000×*g* for 5 min, thus yielding a supernatant and a pellet. The latter was re-homogenized under the same conditions as the first homogenization and recentrifuged at 1000×*g* for 5 min. The supernatants from these two centrifugations were gathered and filtered through four cotton gauze layers and then centrifuged at 10,500×*g* for 10 min to yield the raw mitochondrial pellet that was resuspended in medium A and further centrifuged at 10,500×*g* for 10 m min in to obtain the final mitochondrial pellet. The latter was resuspended by gentle stirring using a Teflon Potter Elvejehm homogenizer in a small volume of medium A, thus obtaining a protein concentration of 30 mg/mL. All steps were carried out at 0–4 °C. The protein concentration was determined according to the colourimetric method of Bradford by the Bio-Rad Protein Assay kit II, using BSA as standard [29]. The mitochondrial preparations were then stored in liquid nitrogen until the evaluation of F_1_F_O_-ATPase activities.

### 2.2 F_1_ domain preparation

Immediately after thawing, swine heart mitochondrial suspensions were diluted with 50 mL of medium An up to obtain a concentration of 20 mg/mL protein, sonicated on ice with MSE Soniprep 150 Sonicator at 210 μm amplitude for 3 min for three times with 30 s intervals, and centrifuged at 10,000×*g* for 10 min. The supernatant from this first centrifugation was further centrifuged at 100,000×*g* for 2 h. All these centrifugation steps were performed at 4 °C. The pellet was resuspended in medium A plus 4 mM Na_2_ATP, the pH was adjusted to 9.2 by the addition of small aliquots of 20 % (w/w) NH_4_OH solution, and stored overnight at 4 °C. Then, the suspension, in which the pH was brought back to 8.0 by adding small aliquots of 2 M HCl aqueous solution, was sonicated at 210 μm amplitude for 5 min. The sonicated suspension was centrifuged at 300,000×*g* for 1 h and the resulting pellet was resuspended in 9 mL medium A plus 2 mM EDTA, pH 7.6. Then, after the addition of 4.5 mL chloroform, the resulting mixture was vigorously vortexed for 15 s and centrifuged at 600×*g* for 10 min to allow the separation of the two phases. The upper aqueous phase was collected and further centrifuged at 100,000×*g* for 1 h. The pale-yellow supernatant obtained was supplemented with adequate aliquots of freshly prepared ATP solution to obtain a final concentration of 4 mM ATP and with 2 M NaOH solution to adjust the pH to 8.0. After the dropwise addition of saturated (NH_4_)_2_SO_4_ solution plus 5 mM EDTA under continuous stirring to obtain 37 % saturation and pH adjustment to 8.0 with 1 M KOH solution, the suspension was centrifuged at 10,000×*g* for 15 min [30]. The pellet was discarded, and the collected supernatant was brought to 60 % saturation with solid (NH_4_)_2_SO_4_; the mixture was then adjusted to pH 8.0 with 1 M KOH solution and kept overnight at 4 °C. Finally, the pellet from the last centrifugation at 150,000×*g* for 90 min, resuspended by gentle stirring using a Teflon Potter Elvehjem homogenizer in a small volume of medium containing 100 mM Tris/H_2_SO_4_, 1 mM EDTA, and 50 % glycerol, pH 8.0, constituted the partially purified F_1_ fraction. The protein concentration was determined according to the colourimetric method of Bradford by Bio-Rad Protein Assay kit II by using BSA as standard [29]. Once verified that in the partially purified F_1_ fraction, the ATPase activity, either sustained by Ca^2+^ or Mg^2+^, was completely insensitive to 1 μg/mL oligomycin, thus proving the detachment of the F_O_ sector, and no further purification was carried out. The partially purified F_1_ fraction was then stored in liquid nitrogen until the evaluation of F_1_-ATPase activities.

### 2.3 Mitochondrial F_1_F_O_-ATPase activity assays

Immediately after thawing, mitochondrial preparations were used to evaluate the F_1_F_O_-ATPase activity. The ATP hydrolysis capability was assayed in a reaction medium (1 mL) containing 0.15 mg mitochondrial protein and 75 mM ethanolammine–HCl buffer (pH 9.0), 6.0 mM Na_2_ATP, and 2.0 mM MgCl_2_ for the Mg^2+^-activated F_1_F_O_-ATPase assay, and in the same buffer at pH 8.8 plus 3.0 mM Na_2_ATP and 2.0 mM CaCl_2_ to evaluate the Ca^2+^-activated F_1_F_O_-ATPase activity. After 5 min preincubation at 37 °C, the reaction, carried out at the same temperature, was started by adding the substrate Na_2_ATP and stopped after 5 min by adding 1 mL of ice-cold 15 % (w/w) trichloroacetic acid (TCA) aqueous solution. Once the reaction was blocked, vials were centrifuged for 15 min at 3500 rpm (Eppendorf Centrifuge 5202). The concentration of inorganic phosphate (Pi) hydrolyzed by known amounts of mitochondrial protein in the supernatant, which indirectly detects the F_1_F_O_-ATPase activity, was spectrophotometrically evaluated. For this purpose, 1.0 μL of 3.0 mg/mL oligomycin in dimethylsulfoxide (DMSO) was directly added to the reaction mixture before starting the reaction. The total ATPase activity was calculated by Pi evaluation in control tubes run in parallel and containing 1.0 μL DMSO per mL reaction system. Control tubes were alternated with the condition to be tested in each set of experiments. The dose of 3.0 mg/mL oligomycin, a specific inhibitor of F_1_F_O_-ATPase that selectively blocks the F_O_ subunit, which is currently used in F_1_F_O_-ATPase assays, ensured maximal F_1_F_O_-ATPase inhibition. The F_1_F_O_-ATPase activity, measured by subtracting the Pi hydrolyzed in the presence of oligomycin from the Pi hydrolyzed by total ATPase activity, was expressed as μmol Pi·mg protein^−1^ min^−1^ in all experiments [31].

### 2.4 F_1_-ATPase activity assays

Immediately after thawing, partially purified F_1_ domains were used for F_1_-ATPase activity assays. The capability of ATP hydrolysis was assayed in a reaction medium (1 mL) containing 0.15 mg F_1_ purified protein and 75 mM ethanolammine–HCl buffer pH 9.0, 6.0 mM Na_2_ATP, and 2.0 mM MgCl_2_ or 2.0 mM CaCl_2_ for the Mg^2+^-activated F_1_F_O_-ATPase and Ca^2+^-activated F_1_F_O_-ATPase assays, respectively. The methods and parameters of ATP hydrolysis and Pi detection were the same as those used for the mitochondrial F_1_F_O_-ATPase activity assays. The sensitivity to 1 μg/mL oligomycin was tested to verify the detachment of F_O_ domain [32].

### 2.5 Arrhenius plots

Arrhenius plots of the Mg-activated F_1_F_O_-ATPase activity on swine heart mitochondria were built to evaluate the temperature dependence of the enzyme properties with and without melatonin. To build such plots, the enzyme-specific activities, evaluated at 4–5 °C intervals in the temperature range 8–37 °C, were taken as the expression of the reaction constant rate *k*. Accordingly, ln *k* was plotted against the reciprocal of the absolute temperature T (in °K), according to the linear form of Arrhenius equation (*i*):

(i) lnk=lnA−EaR1T

where *k* is the rate constant, *A* is the pre-exponential factor, *E*_a_ is the activation energy, *R* is the gas constant and T is the absolute temperature. As expected, as a typical feature of membrane-bound enzymes, two intersecting straight lines were obtained. The activation energies above and below the point of discontinuity (break or melting temperature, Tm) were directly calculated from the slopes of the straight lines obtained, multiplied by the gas constant *R*. According to the units employed, the activation energies were then expressed as kcal/mol. The correlation coefficients, never lower than 0.97, confirmed the linearity of all plots.

### 2.6 Kinetic analyses

To calculate the IC_50_ values, namely the inhibitor concentration which causes half-maximal inhibition of the enzyme activity, the enzyme activity data obtained in the absence of melatonin and in the presence of increasing melatonin concentrations were used to calculate the enzyme inhibition that, after background correction, was fitted to a 3 parameter equation (*ii*), where the lower data limit (no enzyme inhibition) is 0. In equation (ii) the enzyme activity (*y*) is a function of the inhibitor concentration (*x*), “*Range*” is the uninhibited enzyme activity (in the absence of the melatonin), and *s* is a slope factor. As *x* is at the denominator, *y* falls at increasing *x* values.

(ii) $$y=\frac{\text{Range}}{{1+\left(\frac{x}{IC50}\right)}^{s}}$$

The graphical methods of Dixon and Cornish-Bowden plots [33], which complement one another, were used to detect the inhibition mechanism of melatonin on the Ca^2+^-activated F_1_F_O_-ATPase. The 1/*V* (reciprocal of the enzyme activity) in Dixon plot or the S/*V* ratio in Cornish-Bowden plot were plotted as a function of the melatonin concentration. To build these plots, different experimental sets were designed in which the F_1_F_O_-ATPase activity was evaluated in the presence of increasing melatonin concentrations at two ATP concentrations, keeping the cofactor concentration constant. The values of *K'*_i_, which represent the dissociation constant of the ternary *ESI* complex, were calculated as the abscissa (changed to positive) of the intercept of the straight lines obtained in the Cornish-Bowden plots [34]. In all plots, the enzyme-specific activity was taken as the expression of *V*.

Kinetic studies on the mutual exclusion of different inhibitors on the same F_1_F_O_-ATPase activity were carried out. These analyses aimed at identifying a possible interaction on the F_1_ domain between melatonin and 4-Chloro-7-nitrobenzofurazan (NBD-Cl), a known F_1_ inhibitor and on the F_O_ domain between melatonin and DCCD, a known F_O_ inhibitor. To build Dixon-like plots, in which the reciprocal of enzyme activity data (1/*V*) (*y*-axis) was plotted versus melatonin concentration (*x*-axis), the F_1_F_O_-ATPase activity was assayed in the presence of increasing melatonin concentrations at fixed concentrations of F_1_ or F_O_ inhibitor and at constant ATP substrate concentration. According to the graphical method employed [35], when the straight lines show different slopes and intersection points, the enzyme inhibition mirrors the combined effect of the two inhibitors. When the F_1_F_O_-ATPase is inhibited by two not mutually exclusive compounds, for instance melatonin (*I*_*1*_) plus F_1_ or F_O_ inhibitor (*I*_*2*_), the enzyme can combine with both inhibitors yielding the quaternary complex *ESI*_*1*_*I*_*2*_. The value of -α*K'*_i_, which represents the dissociation constant of the quaternary *ESI*_*1*_*I*_*2*_ complex, was calculated from the abscissa (changed to positive) of the point of intersection of the two straight lines obtained in the presence and absence of F_1_ or F_O_ inhibitor. The interaction constant α was then calculated from the ratio of α*K'*_i_ to *K'*_i_.

### 2.7 Oxidative phosphorylation (OXPHOS) assay

Evaluation of oxidative phosphorylation was performed using Seahorse XFp analyzer (Agilent, Santa Clara, CA, USA). 2 μg of freshly extracted and paired mitochondria were loaded into each well of Agilent plate and resuspended in 25 μL of MAS medium (70 mM Sucrose, 220 mM mannitol, 10 mM KH_2_PO_4_, 5 mM MgCl_2_, 2 mM HEPES, 1 mM EGTA and 0.2 % BSA, pH 7.2 with NaOH) implemented with 5 mM Pyruvate/Malate (1: 1) (to assess OXPHOS from the first phosphorylation site) or 10 mM succinate plus 2 μM rotenone (to assess OXPHOS from the second phosphorylation site). After centrifuging at 2,000×*g* for 20 min at 4 °C, 180 μL per well was reached with MAS medium with 1 and 10 mM of melatonin for selected wells. The injection ports of the XFp sensor cartridges were hydrated overnight with the XF calibrator at 37 °C. On the day of analysis, the cartridges were loaded with a concentration of 40 mM ADP in port A, 32 μM oligomycin in port B, 40 μM carbonyl cyanide-4-(trifluoromethoxy) phenylhydrazone (FCCP) in port C and 40 μM Antimycin A in port D. It was possible to obtain the following parameters: baseline OCR detected before addition of ADP (state 2); respiration associated with ATP synthesis recorded after addition of ADP (state 3), OCR in the presence of oligomycin (state 4o) when no ATP is synthesized; maximal respiration stimulates with protonophore after addition of FCCP (state 3u) and OCR after addition of Antimycin A to obtain nonspecific OCR [36,37]. The parameter values, analyzed using WAVE software, were calculated per well on at least three independent experiments and were normalized to the μg protein per well.

### 2.8 mPTP and superoxide anion evaluation

Immediately after the preparation of swine heart mitochondrial fractions, fresh mitochondrial suspensions (1 mg/mL) were energized in the assay buffer (130 mM KCl, 1 mM KH_2_PO_4_, 20 mM HEPES, pH 7.2 with TRIS), incubated at 37 °C with 1 μg/mL rotenone and 5 mM succinate. To evaluate the melatonin effect, 10 mM of it was added to the mitochondrial suspensions before mPTP evaluation. mPTP opening was induced by the addition of low concentrations of Ca^2+^ (10 μM) as CaCl_2_ solution at fixed time intervals (1 min). The calcium retention capacity (CRC), whose lowering indicates mPTP opening, was spectrofluorophotometrically evaluated in the presence of 0.8 μM Fura-FF. The probe has different spectral properties in the absence and presence of Ca^2+^; namely, it displays an excitation/emission spectra of 365/514 nm in the absence of Ca^2^ (Fura-FF low Ca^2+^) and shifts to 339/507 nm in the presence of high Ca^2+^ concentrations (Fura-FF high Ca^2+^). mPTP opening was evaluated by the increase in the fluorescence intensity ratio (Fura-FF high Ca^2+^)/(Fura-FF low Ca^2+^), which indicates a decrease in CRC [38]. All measurements were processed by LabSolutions RF software.

The antioxidant effect of melatonin was evaluated on the superoxide anion (SOX) production in mitochondria, detected by the mitoSOX Red indicator [38]. The production of SOX by mitochondria after the addition of 1 μM Antimycin A (stimulated the SOX generation) was observed as MitoSOX fluorescence intensity (a.u.) increase at absorption/emission spectra of 396/610 nm using the Varioskan™ LUX multimode microplate reader. In detail, 0.2 μg of fresh mitochondrial protein were inserted in black 96-well plates and energized in the buffer assay (130 mM KCl, 1 mM KH_2_PO_4_, 20 mM HEPES, pH 7.2 with TRIS), incubated at 37 °C with 5 mM pyruvate/malate (energized mitochondria with substrates for the first site of phosphorylation) or 5 mM succinate plus 1 μg/mL rotenone (substrate for the second site of phosphorylation). The medium was respectively implemented with 10 μM of MitoSOX probe. To evaluate the melatonin effect, 10 mM of it was added to the mitochondrial suspensions before SOX evaluation. Fluorescence was assessed for 10 min and the measurements were processed by SkanIt RE 7.0.2 software.

### 2.9 Cell cultures

Primary cell cultures of pAECs were isolated, expanded and characterized as previously described [39,40]. pAECs from 3 to 6 passages were used to perform the experiments. The cells were seeded and routinely cultured in T25 or T75 primary culture flasks (2 × 10^4^ cells/cm^2^) in a human endothelial serum-free medium (hESFM), added to a 5 % Fetal Bovine Serum (FBS) and 1 × antibiotic/antimycotic solution in a 5 % CO_2_ atmosphere and at 38.5 °C. An inverted Eclipse Microscope (TS100) with a digital C-Mount Nikon photo camera (TP3100) was used to check cell morphology.

### 2.10 Cell viability

To evaluate the effect of melatonin on cell viability and the protective effect of melatonin on a model of in vitro Hypoxia/Reoxygenation [32], MTT assay was used as previously described [41]. To test the effect of Melatonin on cell culture maintenance: pAECs were cultured on 96 well plate in the presence or absence of increasing Melatonin doses (range from 1 to 25 mM). For H/R model the cells were seeded in 96-well plates at a density of 2x10^4^ cells/well. The day after cell culture medium was replaced by the acid buffer (137 mM NaCl, 12 mM KCl, 0.9 mM CaCl_2_, 0.49 mM MgCl_2_, 4 mM HEPES and 20 mM sodium dl-lactate at pH 6.2) and cells were placed in a modular incubator chamber (Billups-Rothenberg USA) containing a gas mixture (1 % O_2_, 5 % CO_2_, and 94 % N_2_). For 6 h, the normoxic conditions and the complete culture medium were restored for 24 h (reoxygenation) before the cell viability assay, Control group (CTR) was represented by pAECs cultured in normoxic conditions and in cell culture medium with the vehicle (0.01 % DMSO).

### 2.11 Cellular metabolism

Using the Seahorse XFp analyzer (Agilent, Santa Clara, CA, USA), studies of cellular energy metabolism were carried out by measuring the oxygen consumption rate (OCR) and the cellular respiration index (pmol/min). The pAECs (20 × 10^3^/well) were seeded in XFp cell culture mini-plates (Agilent, Santa Clara, CA, USA). The culture medium was replaced with Seahorse XF DMEM medium, pH 7.4, supplemented with 10 mM glucose, 2 mM l-glutamine and 1 mM sodium pyruvate. The analyses were conducted in the absence of Melatonin (control) and in the presence of 1 mM Melatonin for the Mito Stress Test. OCR was measured with the Cell Mito Stress Test program for 45 min at 37 °C. In addition, the injection ports of the XFp sensor cartridges were hydrated overnight with the XF calibrant at 37 °C. On the day of analysis, the cartridges were loaded with 10 times the concentration of inhibitors, as indicated in the instructions for the Cell Mito Stress Test. Final concentrations were 1.5 μM oligomycin (olig) (port A), 1.0 μM FCCP (port B), and 0.5 μM rotenone plus antimycin A (port C). Using the Mito Stress Test, it was possible to obtain information on cellular respiration through the following parameters: basal respiration, the basic OCR detected before the addition of oligomycin; minimal respiration, measured via the OCR in the presence of oligomycin; and maximum respiration, the OCR after the addition of FCCP, as well as the proton leak, which corresponds to the difference between basal respiration and respiration in the presence of oligomycin (minimal respiration), and non-mitochondrial respiration, evaluated in the presence of rotenone plus antimycin A (respiratory chain inhibitors). The latter was subtracted from all the above parameters. ATP production was obtained by assessing the difference between basal respiration and minimal respiration (OCR in the presence of oligomycin), whereas the difference between maximal and basal respiration was used to determine the spare respiratory capacity [42]. The parameter values, analyzed using WAVE software, were calculated per well, according to the manufacturer's instructions, on at least three independent experiments and were normalized to the total number of cells per well.

### 2.12 Superoxide anions detection in cells

The antioxidant effect of melatonin on ROS produced in the H/R model compared to normoxic condition of pAECs was evaluated on SOX detected by the red indicator mitoSOX using the Varioskan™ LUX multimode microplate reader. pAECs (20 × 10^3^/well) were seeded in black 96-well plate. The culture medium was replaced with XF DMEM medium, pH 7.4, supplemented with 10 mM glucose, 2 mM l-glutamine and 1 mM sodium pyruvate implemented with 1 μM MitoSox probe and 1 mM melatonin for selected wells. After 30 min of incubation at 37 °C in the dark, SOX production by mitochondria was recorded as an increase in fluorescence intensity (a.u.) of MitoSOX at absorption/emission spectra of 396/610 nm. All measurements were processed by SkanIt software.

### 2.13 Statistical analysis

All values are expressed as mean ± standard deviation (SD). Comparisons between the experimental groups were performed by one-way ANOVA followed by Dunnet test or T-test. A *P* value of <0.05 was considered significant. GraphPad Prism (Ver 9.1.0 GraphPad Software, Inc., La Jolla, CA, USA) statistical software was used for the statistical analysis.

## 3 Result

### 3.1 Melatonin on F_1_F_O_-ATPase activity and OXPHOS

**Fig. 1:**
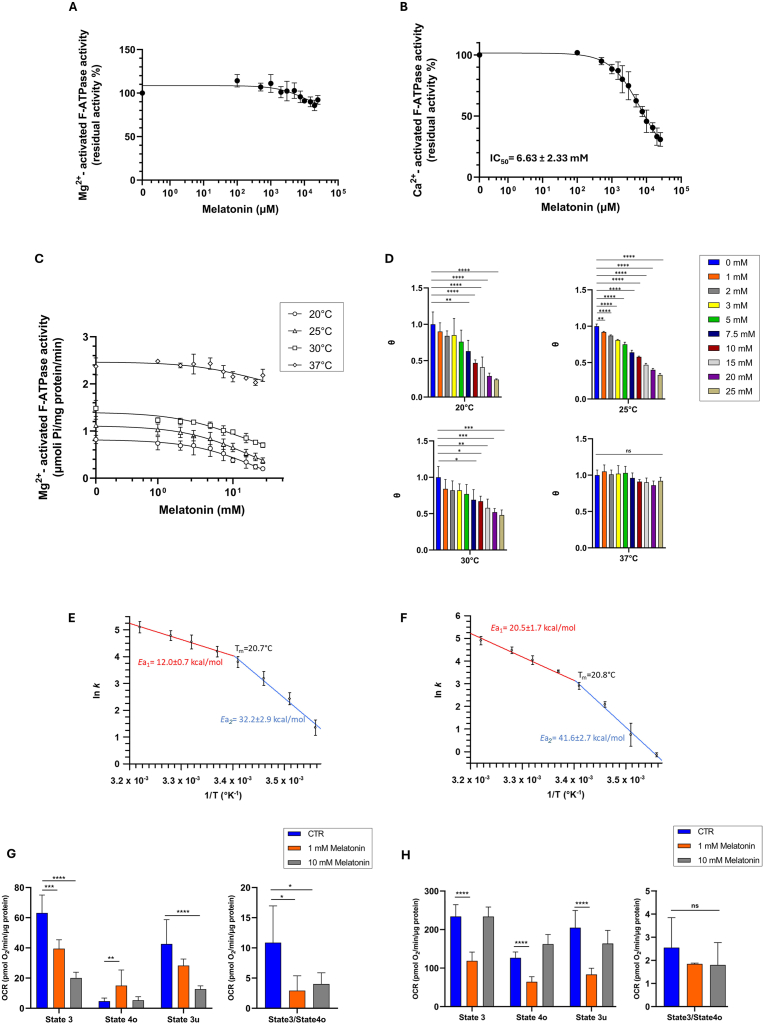
Effect of melatonin on mitochondrial Mg^2+^- and Ca^2+^-activated F_1_F_O_-ATPase activities and OXPHOS. Melatonin titration curve on mitochondrial (A) Mg^2+^- and (B) Ca^2+^-activated F_1_F_O_-ATPase activities at increasing melatonin concentrations. Mg^2+^-activated F_1_F_O_-ATPase activities were evaluated at increasing concentrations of melatonin (1–25 mM) at different temperatures (20–25 - 30–37 °C) (C, D). Arrhenius diagram in the absence (E) and 10 mM melatonin (F). Tm indicates the temperature of the discontinuity (break) point of the diagram; *E*a_1_ red and *E*a_2_ blue indicate the activation energies above and below Tm, respectively. Melatonin effect on selected oxidative phosphorylation parameters: State 3, State 4o, State 3u and State3/State 4o ratio. G) Pyruvate/malate and (H) Succinate-stimulated mitochondrial oxidative phosphorylation without (CTR, ) and with 1 mM () or 10 mM () melatonin. Data represent the mean ± SD (vertical bars) from at least three independent experiments carried out on different mitochondrial preparations. Statistical analysis was performed by Dunnett's test on each group *vs* the control (0 mM melatonin) (D). ∗ Indicate significantly different (∗*P* < 0.05; ∗∗*P* < 0.01; ∗∗∗*P* < 0.001; ∗∗∗∗*P* < 0.0001), ns indicate no significantly different.

The in vitro studies were conducted to evaluate the mechanism of action of melatonin on the mitochondrial enzyme. The concentration used to test the melatonin on isolated mitochondria reflected the possible concentration reached by melatonin in the enzyme microenvironment to exert its biological effect. The dose-response effect of melatonin was evaluated on Mg^2+^- (Fig. 1A) and Ca^2+^- (Fig. 1B) activated F_1_F_O_-ATPase. ATP hydrolysis was differently modulated by melatonin under different conditions of enzyme activation. In the range of 0.1–25 mM, melatonin showed an inhibitory efficiency only on Ca^2+^-activated F_1_F_O_-ATPase but not on Mg^2+^-activated F_1_F_O_-ATPase. For the latter, it was not possible to calculate an IC_50_ value (Fig. 1A). The maximum concentration of melatonin tested (25 mM) inhibited Ca^2+^-activated F_1_F_O_-ATPase by 69 % with an IC_50_ value of 6.63 ± 2.33 mM (Fig. 1B).

The Mg^2+^-activated F_1_F_O_-ATPase activity was evaluated at different temperatures (20–25 – 30–37 °C) (Fig. 1C) to validate the lack of effect by melatonin. The inhibitory effect of melatonin, in the range of 1–25 mM, was temperature-dependent at values of 20–25 – 30 °C. Conversely, the effect was not detected at 37 °C (Fig. 1D).

The temperature-dependent effect was further investigated since biophysical differences in the membrane at different temperatures could contribute to influencing the catalytic activity of the enzyme. A study on Mg^2+^-activated F_1_F_O_-ATPase was performed using the Arrhenius plot in the absence and the presence of 10 mM melatonin, the arbitrary concentration used to study the in vitro effect on mitochondria. Since the Arrhenius plots were discontinuous, two distinct activation energies (*E*a_1_ and *E*a_2_) were calculated above and below the break. Indeed, the discontinuous Arrhenius plots showed a Tm of approximately 20.7 °C or 20.8 °C in the absence of melatonin (Fig. 1E) or in the presence of melatonin (Fig. 1F), respectively. The activation energies of an enzyme reaction, obtained from the slopes above and below the so-called temperature of discontinuity, which is currently taken as correspondent to Tm when an abrupt change in membrane physical state occurs, are associated with efficient enzyme catalysis. Low activation energy is associated with a lower energy barrier that must be overcome to yield the product to an enzyme reaction. When the activation energies of Mg^2+^-activated F_1_F_O_-ATPase activity in the absence of melatonin were compared with that obtained with 10 mM melatonin, they were lower both above (*E*a_1_) and below (*E*a_2_) the Tm. In detail, fast enzymatic reactions with low *E*a values (without melatonin) were slowed down in the presence of melatonin (Fig. 1E and F).

To understand the effect of melatonin on oxidative phosphorylation, mitochondrial respiration coupled to ATP synthesis was assessed in the presence of pyruvate/malate (Fig. 1G) and succinate (Fig. 1H) as substrates for the first and second phosphorylation sites, respectively. The results obtained in the presence of NAD-dependent substrates (first phosphorylation site) (Fig. 1G) in the presence of 1 mM or 10 mM melatonin highlighted that State 3 (a condition in which ATP is synthesized after the addition of ADP) was significantly reduced compared to the control (without melatonin). State 4 (calculated after the addition of oligomycin) was enhanced with 1 mM melatonin, whereas state 3u (evaluated after the addition of the uncoupler FCCP) was significantly reduced only with 10 mM melatonin. Consequently, the State 3/State 4o ratio (indicative of coupling between pyruvate/malate substrate oxidation and ADP phosphorylation) was negatively modified by melatonin at each concentration tested if compared to the control. In the presence of succinate (second phosphorylation site) (Fig. 1H) only 1 mM melatonin showed an inhibitory effect on State 3, State 4o, and State 3u if compared to the control. However, the coupling index between succinate substrate oxidation and ADP phosphorylation was not changed by melatonin.

### 3.2 Inhibition mechanism and multiple inhibition analysis to identify the melatonin interaction site

**Fig. 2:**
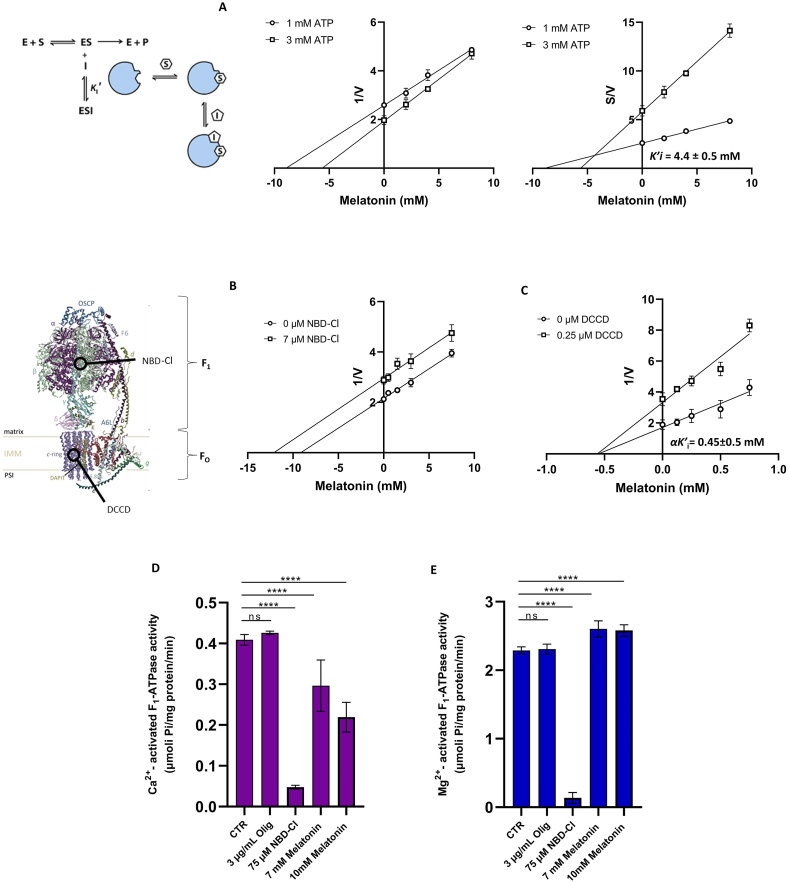
Evaluation of the inhibition mechanism and interaction site of melatonin. A) Inhibition mechanism of melatonin on mitochondrial Ca^2+^-activated F_1_F_O_-ATPase. Dixon (1/V y-axis) and Cornish–Bowden (*S*/*V y*-axis) plots were obtained at 1 mM (○) or 3 mM (□) ATP. Multiple inhibitor analysis using Dixon plots to evaluate the melatonin inhibition on mitochondrial Ca^2+^-activated F_1_F_O_-ATPase. The activity was assessed in the absence (○) or presence of 7 μM NBD-Cl (□) (B); in the absence (○) or presence of 0.25 μM DCCD (□) (C). Effect of melatonin on the F_1_ domain (D, E). The activities of F_1_-ATPase activated by (D) Ca^2+^ and (E) Mg^2+^ were evaluated in the absence or presence of inhibitors: 3 μg/mL oligomycin; 7 mM or 10 mM melatonin, or 75 μM NBD-Cl. All points represent the mean ± SD (vertical bars) of three separate experiments performed on different mitochondrial preparations. Statistical analysis was performed by Dunnett's test on each group vs the control (CTR) (D, E). ∗ Indicate significantly different (∗∗∗∗*P* < 0.0001), ns indicate no significant difference.

The mechanism of melatonin inhibition on Ca^2+^-activated F_1_F_O_-ATPase was evaluated by Dixon and Cornish-Bowden plots. Melatonin inhibition was uncompetitive towards the ATP substrate (Fig. 2A). Melatonin binds to the enzyme already complexed with ATP (*ES*) forming the tertiary complex (*ESI*) (Fig. 1A). Furthermore, the Cornish-Bowden plot has been used to obtain the dissociation constant of the *ESI* complex (*K'*i = 4.4 ± 0.5 mM) of Ca^2+^-activated F_1_F_O_-ATPase.

Mutual exclusion analyses, performed by incorporating binary mixtures of F_1_ and F_O_ inhibitors into the F-ATPase reaction medium, aimed to verify the possible interaction of melatonin with the catalytic sites of the hydrophilic F_1_ domain (NBD-Cl (*I*_1_) plus melatonin (*I*_2_)) (Fig. 2B) or with the F_O_ portion (DCCD (*I*_1_) plus melatonin (*I*_2_)) (Fig. 2C). These experiments aimed to clarify whether melatonin can combine with the ternary *ESI*_*1*_ complex to form the quaternary *ESI*_*1*_*I*_*2*_ complex or whether the binding of *I*_2_ prevents the binding of melatonin, in other words, whether the compounds tested on F_1_F_O_-ATPase are mutually exclusive. The reciprocal of the Ca^2+^-activated F_1_F_O_-ATPase activity in the presence and absence of 7 μM NBD-Cl was plotted as a function of increasing melatonin concentrations. Two straight parallel lines were obtained (Fig. 2B). This result depicted an exclusive interaction of melatonin or NBD-Cl with the enzyme. Similarly, using increasing concentrations of melatonin in the absence and presence of 0.25 μM DCCD, evaluating the Ca^2+^-activated F_1_F_O_-ATPase activities, two straight lines intersected on the *x*-axis were obtained (Fig. 2C). This result highlighted a simultaneous interaction of melatonin with DCCD on the enzyme. The α*K*′i value obtained graphically represented the dissociation constant of the *ES∙DCCD∙melatonin* (*ESI*_*1*_*I*_*2*_) complex. The interaction constant (α) between two different compounds bound to the enzyme obtained from the ratio α*K*′i to *K*′i indicated whether the binding of the inhibitor (DCCD) influenced (α≠1) or did not influence (α = 1) the binding of melatonin to the *ES* complex. Since a value of α < 1 was obtained (α = 0.1), the binding of one inhibitor favoured the interaction of the other inhibitor.

To verify that the melatonin binding site was located on the F_1_ domain, its effect on the purified F_1_ catalytic portion, which independently performs ATP hydrolysis, was assessed. F_1_-ATPase activity was insensitive to oligomycin and was inhibited by NBD-Cl. The inhibitors were specific inhibitors of the F_O_ or F_1_ domain, respectively, independently if the enzyme catalysis was tested with Ca^2+^ (Fig. 2D) or Mg^2+^ (Fig. 2E) as cofactor on the purified portion F_1_. The data showed the inhibitory effect exerted by melatonin on Ca^2+^-activated F_1_-ATPase (Fig. 2D) without affecting Mg^2+^-activated F_1_-ATPase. The results were consistent with what occurred on the Ca^2+^- and Mg^2+^-activated F_1_F_O_-ATPase (Fig. 1A and B).

### 3.3 mPTP modulation and antioxidant effect of melatonin

**Fig. 3:**
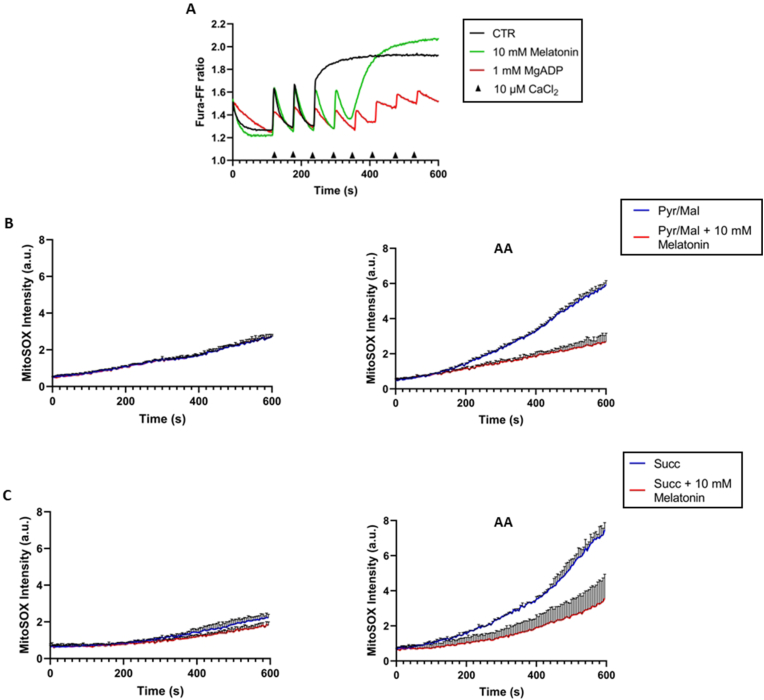
Evaluation of mPTP opening and ROS production on isolated mitochondria. A) Representative curves of four experiments of calcium retention capacity (CRC). CRC was monitored in response to successive pulses of 10 μM CaCl_2_ (shown by arrows), in the absence (CTR-black line) and in the presence of the inhibitor 1 mM MgADP (red line) and 10 mM melatonin (green line). B) Evaluation of superoxide anion production in mitochondria energized with pyruvate plus malate as substrates for the first oxidative phosphorylation site (complex I), and with succinate (C) as substrate for the second oxidative phosphorylation site (complex II). The red line indicates the presence of 10 mM melatonin. Graphs labeled AA indicate the preliminary addition of 1 μM antimycin A to mitochondrial respiration to trigger superoxide anion stimulation. Experiments were performed in triplicate on three separate mitochondrial preparations. All points represent the mean ± SD (vertical bars) of three separate experiments performed on different mitochondrial preparations.

mPTP opening was assessed by studying the CRC, as well as the ability of intact mitochondria to accumulate Ca^2+^. The latter accumulated in the mitochondrial matrix and was released when the mPTP opened. Shown as the ratio (Fura-FF high Ca^2+^)/(Fura-FF low Ca^2+^), the decrease in CRC in melatonin-treated mitochondria, *i.e.*, a greater number of calcium pulses to induce mPTP opening compared to the control, highlighted the ability of melatonin to desensitize mPTP opening (Fig. 3A). Therefore, mitochondria in the presence of melatonin must reach a higher threshold value of Ca^2+^ concentration in the matrix to trigger mPTP formation, compared to control condition.

Antimycin A (AA) induced SOX production on isolated mitochondria was monitored as the fluorescence intensity of the MitoSox probe. By energizing mitochondria with the addition of substrates pyruvate/malate to the phosphorylation site I, 10 mM melatonin completely abolished AA-induced ROS levels, as much as the control values assessed without AA stimulation (Fig. 3B). Similarly, melatonin abolished SOX reduction by energizing mitochondria from second site of phosphorylation, *i.e.*, in the presence of mitochondria energized with substrate succinate and rotenone (Fig. 3C).

### 3.4 Melatonin on pAECs’ H/R model

**Fig. 4:**
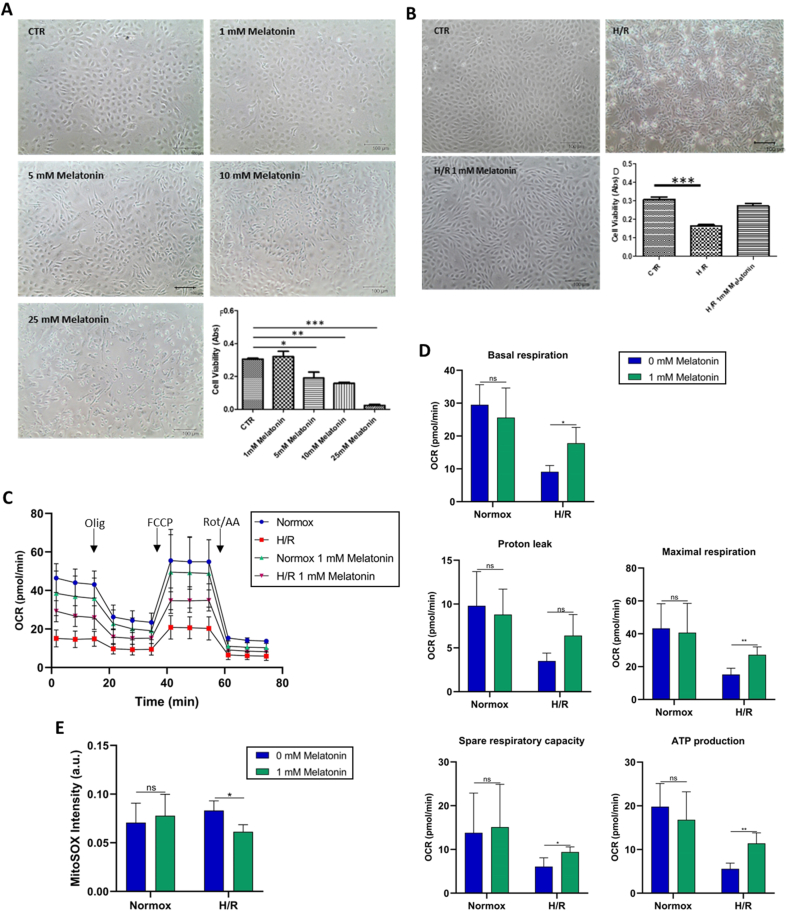
Effect of melatonin on pAECs under H/R condition. A) Representative images of pAECs treated with 0, 1, 5, 10, or 25 mM of melatonin. B) Effect of melatonin on H/R injury model. Representative images of pAECs after H/R injury in the absence or presence of 1 mM melatonin. Each bar represents the mean ± SD of three independent experiments. Scale bar (−) 100 μm. Statistical analysis was performed by one-way ANOVA, post hoc Dunnet comparison test between each Melatonin treatment *vs* the control (CTR) group. ∗ Indicate significantly different (∗*P* < 0.05, ∗∗*P* < 0.01, ∗∗∗*P* < 0.001). C) Effect of melatonin on pAECs metabolism subjected to H/R injury. Mitochondrial respiration profile was obtained from oxygen consumption rate (OCR) in normoxia, without () and with () 1 mM melatonin and in H/R, without () and with () 1 mM Melatonin under basal respiration conditions and after addition of 1.5 μM oligomycin (olig), 1.0 μM FCCP and a mixture of 0.5 μM rotenone plus antimycin A (Rot + AA). Modulator injections are shown with arrows. D) Mitochondrial parameters (basal respiration, proton leak, maximal respiration, spare respiratory capacity and ATP production) in normoxia and H/R without () or in the presence () of 1 mM melatonin. E) Evaluation of superoxide anion production in pAECs in the Normoxia or H/R injury without () or in the presence () of 1 mM melatonin. Each bar represents the mean ± SD of four (D) and three (E) independent experiments. Statistical analysis was performed by Dunnett's test on each group vs the control (0 mM melatonin). ∗ Indicate significantly different (∗*P* < 0.05, ∗∗*P* < 0.01), ns indicate no significant difference.

pAECs’ viability was affected by the presence of melatonin, in particular starting at 5 mM dose, melatonin resulted cytotoxic for endothelial cells losing their typical morphology as a compact monolayer (Fig. 4A). Therefore, for the H/R model, 1 mM was chosen to test melatonin protective effect on the H/R injury model. In particular, after H/R treatment cells appeared detached losing their typical phenotype, the presence of melatonin during H/R injury restored the adherent monolayer and the cell viability was not different from control cells in normoxia (Fig. 4B).

To verify the metabolic remodelling of pAECs by melatonin, mitochondrial bioenergetic metabolism analyses were performed (Fig. 4C and D). The cellular respiration profile of cells treated with and without 1 mM melatonin (Fig. 4C) showed a functional rescue of cellular respiration also under H/R condition. The parameters of mitochondrial activities were recorded as: basal OCR before the addition of oligomycin; proton leak, which corresponded to the difference between the basal respiration and the respiration measured as OCR in the presence of oligomycin (minimal respiration); the maximum respiration, assessed as OCR after the addition of FCCP; the spare respiratory capacity provided by the difference between maximum and basal respiration; and the ATP production, has been evaluated from the difference between the basal respiration and the minimal respiration (OCR in presence of oligomycin). All parameters were not affected by melatonin in normoxia, whereas we detected recovery of the OCR decline caused by H/R in pAECs treated with melatonin in all bioenergetic parameters except for the proton leak which was not affected (Fig. 4D).

SOX production induced by H/R injury on pAECs compared to normoxia condition was monitored as fluorescence intensity of the MitoSox probe. Treatment with 1 mM melatonin significantly reduced H/R-induced SOX levels (Fig. 4E).

## 4 Discussion

Recent studies report that melatonin has a clear effect on mitochondrial quality control, causing a profound reprogramming of cellular metabolism through actions at different mitochondrial levels [43]. Our study shows that melatonin has a specific mitochondrial molecular target, namely the hydrophilic portion of the F_1_F_O_-ATPase enzyme. Its binding to the F_1_ portion was confirmed by *i*) mutual exclusion studies with NBD-Cl, known to inhibit F_1_F_O_-ATPase activity by interacting with an amino acid residue of β subunit in empty conformation in the F_1_ domain; *ii*) specific inhibitor effect on the hydrolytic activity of the purified F_1_ portion [31,44]. However, the melatonin-enzyme interaction modulated the F_1_F_O_-ATPase activity in a Ca^2+^-dependent manner without affecting the ATPase activity of the enzyme when activated by natural cofactor Mg^2+^ at 37 °C. This suggested a targeted action of melatonin on Ca^2+^-dependent regulatory mechanism of the F_1_F_O_-ATPase. Interestingly, Mg^2+^-activated F_1_F_O_-ATPase inhibition occurred only at low temperatures, which may indicate a thermal sensitivity in the conformation or binding of the melatonin to the Mg^2+^-dependent F_1_F_O_-ATPase [45].

The inhibition of Mg^2+^-activated F_1_F_O_-ATPase at low temperatures was not due to an effect of the melatonin on the fluidity of the mitochondrial membrane. Indeed, the Tm values were the same with and without melatonin. Accordingly, the break constancy and the concomitant increase in both activation energies above and below the break in the presence of melatonin might be interpreted in terms of antagonistic compounds of catalytic efficiency. By excluding a direct effect on the lipid structure of the membrane, it was possible to confirm that the main target of melatonin was the enzyme and not the dynamics of the membrane itself.

A further inhibitory effect of melatonin was found in the process of oxidative phosphorylation, on the coupling index. The adverse action on ATP phosphorylation coupled to the oxidation of NAD-dependent substrates was more marked when mitochondrial respiration was stimulated with the first phosphorylation site, using pyruvate/malate as a substrate. This highlighted a specificity towards complex I of the mitochondrial respiratory chain, which could be a key point for modulating energy production. Moreover, ATP synthesis could suffer the outcome of melatonin's role on the electron transport chain connected to oxidative phosphorylation linked to its capacity to trap electrons [46]. Research involving isolated mitochondria has the benefit of controlled conditions to analyze direct chemical interactions, but it is entirely devoid of the complex regulatory networks seen in the cellular environment. The difference between working with isolated mitochondria and intact cells revealed that melatonin did not reduce cell respiration. Performing studies of the mechanisms on mitochondria, without interference from cytosolic factors, allowed for monitoring of mitochondrial bioenergetics function with higher precision. Conversely, in pAECs the interaction with the rest of the cell was preserved and, overriding the complexity of system biology, physiological relevance was greatly enhanced and the mitochondrial environment was exposed to a relevant mix of substrates/ions and other organelles and cell structures [47]. The analysis about the molecular mechanism of melatonin in isolated mitochondria has provided essential bioenergetics features for the interpretation of oxidative metabolism in pAECs. Our investigations revealed an interaction site between melatonin and F_1_F_O_-ATPase in isolated mitochondria when the enzyme was activated by Ca^2+^, concomitant with mPTP inhibition and a decrease in ROS levels. The observed effects of melatonin on isolated mitochondria could provide evidence supporting the protective mechanisms of melatonin in pAECs exposed to H/R.

Cell metabolism of pAECs evaluated at no toxic concentration of melatonin (Fig. 4A) was not responsible for the decrease in cell respiration (Fig. 4D). Noteworthy is the ability of melatonin to inhibit the mPTP opening. The mPTP phenomenon is associated with cell death such as necrosis or apoptosis [48,49]. This property could represent a potential therapeutic application, especially in conditions of mitochondrial stress. Indeed, the decrease in the production of ROS was also supported by the protective effect of melatonin revealing a particularly useful feature in pathological conditions associated with oxidative stress [50,51]. In addition to this, previous studies have shown that mitochondrial complex III inhibition leads to an increase in reduced CoQ (CoQH_2_) leading to reverse electron transport from CoQH_2_ to complex I, and a resulting generation of SOX [52]. In this process of ROS generation, melatonin could act as a free radical scavenger, an effect that may account for some of the protective properties of its indoleamine structure [53] under pathological conditions [54,55]. Counteraction of the oxidative stress in mitochondria provides evidence of a new hormonal mechanism regulating the redox homeostasis in mitochondria by performing ATP production [46]. The underlying mechanism of melatonin was also linked to reprogramming cancer cell metabolism of lung cancer cells. Melatonin can include a change from cytosolic aerobic glycolysis to oxidative phosphorylation. Beneficial changes were mediated by the activation of Sirtuin 3 participating in ATP production by regulating the acetylone [56]. Moreover, neurotoxicity caused by cadmium drives the disruption of mitochondrial dynamics, particularly by excessive mitochondrial fission. Melatonin has a neuroprotective action on cadmium's effects by restoring the balance between mitochondrial fusion and fission. This protective effect is likely achieved by preventing calcium overload, which in turn blocks the recruitment of Drp1 to mitochondria [57]. On balance, since melatonin has been shown to boost mitochondrial activity in various contexts [46,[53], [54], [55], [56], [57]], it is plausible that it does not negatively impact F_1_F_O_-ATPase in healthy cells. The ability to inhibit the mPTP opening and reduce oxidative stress represent crucial mechanisms to prevent mitochondrial dysfunction, especially in pathological conditions such as I/R and metabolic disorders [58,59].

Mitochondrial dysfunctions arising after the damage induced by the H/R process on pAECs have been evaluated through the parameters of basal respiration, maximal respiration, respiratory capacity, ATP production and mitochondrial SOX generation. Melatonin-dependent protection can rely on its antioxidant action. The decrease of mitoSOX signal in H/R conditions (Fig. 4E) was also supported by the protective SOX production in isolated mitochondria (Fig. 3B and C). The improvement of mitochondrial functions induced by melatonin under stress conditions, such as hypoxia followed by reoxygenation, underlined the potential of the molecule to protect cells in situations of ischemic or metabolic stress. We could assert that melatonin prevented the processes that induced cellular damage, as we have seen by reducing oxidative stress and blocking the mPTP opening, a key event in cell death [47]. It is known that direct inhibitors of the mPTP can mitigate mitochondrial dysfunction induced by the above-mentioned factors responsible for impairing mitochondrial bioenergetics. Since F_1_F_O_-ATPase activated by Ca^2+^ but not in the presence of the natural cofactor Mg^2+^ (Fig. 2D and E) may be a possible molecular target of melatonin and a component of mPTP formation, this could be a further reason for the protective action of melatonin on cell death from H/R injury [60,61]. Molecules that act by fine-tuning the parameters of mitochondrial respiration and reducing oxidative stress, preserve the functional integrity of the inner membrane improving the tolerance of cells to ischemic damage. Therefore, targeting the molecular structure characterising the mPTP may be healthful in related pathophysiological conditions, such as in the context of heart attack and stroke [[48], [49], [50], [51]]. Ultimately, the protective effects exerted by melatonin improving cell survival in H/R conditions opens interesting perspectives for therapeutic applications under conditions of inadequate mitochondrial function.

## 5 Conclusion

Melatonin showed a promising profile as a mitochondrial modulator. The specific effects on mitochondrial Ca^2+^-activated F_1_Fo-ATPase regulated the cell energy metabolism impaired in H/R conditions and protected against oxidative damage induced by mitochondrial ROS production. This made it potentially useful in therapeutic settings such as ischemia-reperfusion, metabolic diseases or other mitochondrial dysfunction conditions involving mPTP opening and mitochondrial SOX overproduction. Melatonin supplementation, therefore, could potentially be considered in clinical applications, although further studies will be needed to better understand the mechanisms of action, safety and efficacy in disease models involving mitochondrial dysfunction underlying pathogenesis such as CVDs.

## CRediT authorship contribution statement

**Cristina Algieri:** Writing – original draft, Validation, Investigation, Formal analysis. **Chiara Bernardini:** Formal analysis. **Antonia Cugliari:** Formal analysis. **Silvia Granata:** Investigation. **Fabiana Trombetti:** Writing – review & editing. **Patrycja Anna Glogowski:** Data curation. **Micaela Fabbri:** Resources. **Giampaolo Morciano:** Writing – review & editing. **Gaia Pedriali:** Writing – review & editing. **Paolo Pinton:** Writing – review & editing, Validation. **Salvatore Nesci:** Writing – original draft, Visualization, Supervision, Funding acquisition, Conceptualization.

## Funding

The research leading to these results received funding from 10.13039/501100000780European Union - Next Generation EU, Missione 4 Componente 1 CUP J53D23008970006, Progetto PRIN 2022 UNDER40 (no. 2022E75TWB_001) to SN. SG is funded by the 10.13039/501100000780European Union - Next Generation EU - PNRR M6C2 - Investimento 2.1 Valorizzazione e potenziamento della ricerca biomedica del SSN, PNRR-MAD-2022-12376295, CUP: F33C22001010006. GM is supported by the 10.13039/501100003196Italian Ministry of Health grant GR‐2019‐12369862.

## Declaration of competing interest

The authors declare the following financial interests/personal relationships which may be considered as potential competing interests:Salvatore Nesci reports financial support was provided by 10.13039/501100005969European Union - Next Generation EU, M4C1, Progetto PRIN 2022 UNDER40 (MUR). If there are other authors, they declare that they have no known competing financial interests or personal relationships that could have appeared to influence the work reported in this paper.

## Data Availability

Data are available on AMSActa Institutional Research Repository by AlmaDL University of Bologna Digital Library https://doi.org/10.6092/unibo/amsacta/8126.
